# Clinical significance of esophageal invasion length for the prediction of mediastinal lymph node metastasis in Siewert type II adenocarcinoma: A retrospective single‐institution study

**DOI:** 10.1002/ags3.12069

**Published:** 2018-04-10

**Authors:** Kazuo Koyanagi, Fumihiko Kato, Jun Kanamori, Hiroyuki Daiko, Soji Ozawa, Yuji Tachimori

**Affiliations:** ^1^ Department of Esophageal Surgery National Cancer Center Hospital Tokyo Japan; ^2^ Department of Gastroenterological Surgery Tokai University School of Medicine Isehara Japan; ^3^ Cancer Care Center Kawasaki Saiwai Hospital Kawasaki Japan

**Keywords:** adenocarcinoma, esophageal invasion length, esophagogastric junction, mediastinal lymph node metastasis, Siewert type II

## Abstract

**Aim:**

This study investigated whether esophageal invasion length (EIL) of a tumor from the esophagogastric junction could be a possible indicator of mediastinal lymph node metastasis and survival in patients with Siewert type II adenocarcinoma.

**Methods:**

One hundred and sixty‐eight patients with Siewert type II tumor who underwent surgery were enrolled. Metastatic stations and recurrent lymph node sites were classified into cervical, upper/middle/lower mediastinal, and abdominal zones. EIL was correlated with overall metastasis or recurrence in individual zones and with survival.

**Results:**

Siewert type II patients with an EIL of more than 25 mm (>25 mm EIL group) had a higher incidence of overall metastasis or recurrence in the upper and middle mediastinal zones than those with an EIL of less than or equal to 25 mm (≤25 mm EIL group) (*P* = .001 and *P* < .001). Disease‐free and overall survival in the >25 mm EIL group were significantly lower than those of the ≤25 mm EIL group (*P* < .001). None of the Siewert type II patients with metastasis or recurrence in the upper and middle mediastinal zones survived for more than 5 years. Only an EIL of more than 25 mm was a significant preoperative predictor of overall metastasis or recurrence in the upper and middle mediastinal zones (odds ratio, 8.85; 95% CI, 2.31‐33.3; *P* = .001).

**Conclusion:**

A multimodal‐therapeutic strategy should be investigated in Siewert type II patients once the tumor has invaded more than 25 mm to the esophageal wall.

## 1 INTRODUCTION

The worldwide incidence of adenocarcinoma of the esophagogastric junction (EGJ) has been increasing in the past few decades.^1^, ^2^, ^3^ The Siewert classification divides this entity into three subtypes according to its anatomical localization relative to the EGJ.^4^ Siewert type I and type III tumors are usually treated as esophageal and gastric tumors, respectively. Because of the higher risk of mediastinal lymph node metastasis, a transthoracic and abdominal approach is frequently used for Siewert type I tumors, whereas an abdominal or transhiatal approach is commonly proposed for Siewert type III tumors. In contrast, Siewert type II tumors are considered “true” EGJ adenocarcinomas.^5^ However, the optimal surgical approach for this tumor remains unclear. Although dissection of the lower mediastinal lymph nodes can be carried out by either a transthoracic or a transhiatal approach, only a transthoracic method can be used to dissect the upper and middle mediastinal lymph nodes. As an R0 resection is essential for long‐term survival after surgery for EGJ adenocarcinoma,^6^, ^7^ the extent of lymph node dissection should be considered as well as the extent of esophageal or gastric resection in individual Siewert type II tumors.

Lymph node metastasis is known as a prognostic factor of esophageal cancer. Previous reports have indicated that a thorough lymph node dissection improves the survival of patients after esophagectomy.^8^, ^9^ However, the accuracy of preoperative assessments of positive lymph node metastasis in patients with EGJ adenocarcinoma using conventional diagnostic techniques, such as computed tomography (CT) and endoscopic ultrasonography (EUS), is not yet as high as needed.^10^ Moreover, given the frequent discrepancy between clinical diagnosis and pathological findings, improvements in the preoperative assessment of positive lymph node metastasis, especially mediastinal lymph node metastasis, are key to optimizing the therapeutic strategy for Siewert type II tumors. Several pathological parameters have been shown to be risk factors for lymph node metastasis in patients with Siewert type II tumors.^11^, ^12^, ^13^, ^14^ However, because neoadjuvant chemotherapy or chemoradiotherapy has been introduced as a standard therapy for resectable EGJ adenocarcinoma,^15^, ^16^ a new clinical indicator that predicts mediastinal lymph node metastasis prior to treatment needs to be investigated for the prompt selection of surgical strategies, including the extent of lymph node dissection.

The surgical strategy for Siewert type II tumors is mainly determined by tumor invasion of the esophageal wall and the relative risk of upper and middle mediastinal lymph node metastasis.^17^, ^18^ Longitudinal lymphatic flow is very rich in the submucosal layer of the esophageal wall;^19^, ^20^ however, the incidence of mediastinal lymph node metastasis is very low when the tumor is predominantly located in the abdominal cavity and has not massively invaded the thoracic cavity.^21^, ^22^ Accordingly, we focused on esophageal invasion length (EIL), defined as the distance from the EGJ to the proximal edge, of Siewert type II tumors and hypothesized that the EIL could be a possible indicator of mediastinal lymph node metastasis in Siewert type II tumors. In the present study, we correlated mediastinal lymph node metastasis or recurrence with EIL in Siewert type II tumors. Also, we assessed the clinicopathological factors and survival according to EIL to investigate the biological significance of EIL in Siewert type II tumors.

## 2 METHODS

### 2.1 Patients

A total of 209 consecutive patients with a clinical diagnosis of Siewert type II adenocarcinoma who underwent surgery at the National Cancer Center Hospital between January 2001 and October 2016 were retrospectively selected. Among them, 11 patients were excluded because of the diagnosis of other histopathological types after surgery. To compare clinical EIL with lymph node metastasis in the study, 18 patients who did not have their EIL assessed prior to surgery and 12 patients who received neoadjuvant chemotherapies were also excluded from the study. Accordingly, a total of 168 patients with Siewert type II tumors were included in further analyses. The present study was approved by the Institutional Review Board, and individual consent to participate in the study was obtained.

### 2.2 Tumor classification

Pretreatment clinical evaluations included esophagogastric endoscopy, esophagogastrography, EUS, CT scans of the neck, chest, and abdomen, and positron emission tomography (PET) if needed. Cancer stage was determined according to the 7th edition of the International Union Against Cancer TNM Classification of Malignant Tumors (UICC 2009).^23^ The EGJ was defined as the distal end of the lower esophageal palisade vessels or as the upper ends of the gastric mucosal folds by endoscopic examination and was defined as the narrowest locus of the lower esophagus by esophagogastrography. The EIL was subsequently determined as the distance from the EGJ to the proximal edge of the tumor. In some advanced circumferential tumors, the EIL was determined from the findings obtained by both endoscopy and esophagogastrography: observation of the cardia from the stomach and His angle. The Siewert classification was determined according to the dominant area of tumor invasion and the epicenter of the tumor.

Areas of lymph node metastases were recorded according to the lymph node stations adopted by the Japanese classifications.^24^ Both the metastatic stations and the recurrent lymph node sites were classified into five lymph node zones: cervical, upper mediastinal, middle mediastinal, lower mediastinal, and abdominal.^25^ The middle mediastinal zone and lower mediastinal zone were divided by the caudal margin of the inferior pulmonary vein. When either a metastasis or a recurrence was detected in any lymph node of each zone, that lymph node zone was considered to be positive for tumor involvement.

### 2.3 Surgical procedures and postoperative surveillance

Selection of surgical procedure was not specified in the present study. Three different approaches were adopted based on the results of preoperative tumor examinations: a right‐sided transthoracic and abdominal approach (RTT), a left‐sided thoracoabdominal approach (LTA), and a transhiatal via abdominal approach (TH). Generally, RTT was selected for advanced tumors or tumors with clinical lymph node metastasis. LTA and TH were selected for mainly superficial tumors, and left thoracic incision was carried out to secure the proximal surgical margin. Extent of lymph node dissection basically depended on the surgical approach: three‐ or two‐field lymph node dissection was used for an RTT, lower to middle mediastinal and abdominal lymph node dissection was used for an LTA, and abdominal and partial lower mediastinal lymph node dissection was used for a TH. The gastric tube was preferably used for reconstruction when an RTT was carried out, and a jejunal interposition was selected when an LTA or a TH was carried out. Respiratory complications included pneumonia, atelectasis, and empyema. The clinical course after surgery was followed every 3 months for the first year, and then every 6 months up to 5 years. In addition to the physical examination and serum tumor markers, CT scan was carried out every 6 months, and endoscopy was carried out once per year to survey tumor recurrence.

### 2.4 Statistical analysis

Chi‐squared test and Fisher's exact test were used to assess the categorical variables, and a *t* test and a Mann‐Whitney *U* test were used to assess continuous variables. Survival curves were generated using the Kaplan‐Meier method, and a log‐rank test was used to compare survival. Receiver operating characteristic (ROC) curves were generated to decide the cut‐off value for the EIL in Siewert type II tumors to predict the risk of mediastinal lymph node metastasis or recurrence. Using the frequency of overall metastasis or recurrence and the 5‐year overall survival (OS) in each zone, an efficacy index was established to evaluate the benefits of mediastinal lymph node dissection in patients with Siewert type II tumor.^25^ A multivariate logistic regression analysis was carried out for the selection of predictive clinical variables that affected upper and middle mediastinal lymph node metastasis or recurrence. The model did not include variables in which the *P*‐value was not significant in a univariate analysis and in which multicollinearity with the EIL was recognized. The analysis was carried out using SPSS software (version 22; SAS Institute, Cary, NC, USA), and the tests were two‐sided with a significance level <.05.

## 3 RESULTS

### 3.1 Characteristics of patients with Siewert type II adenocarcinoma

Characteristics of patients with Siewert type II tumors are summarized in Table 1. There were 63, 21, and 84 patients categorized as having clinical T1, T2, and T3 tumors, respectively. Also, there were 102, 41, 24, and one patients categorized as having clinical N0, N1, N2, and N3 lymph node metastasis, respectively. One patient had supraclavicular lymph node metastasis. Based on the diagnosis of these categories, patients were clinically classified as belonging to stage IA (n = 61), IB (n = 15), IIA (n = 25), IIB (n = 7), IIIA (n = 35), IIIB (n = 23), IIIC (n = 1), and IV (n = 1). Pathological lymph node metastasis was found in 88 (52.4%) patients with Siewert type II tumors. Rate of pathological lymph node metastasis was higher than the rate of clinical lymph node metastasis. Incidence of any lymph node recurrence was 8.9% in Siewert type II tumors. However, as a result of the different rate of mediastinal lymph node dissection, immediate comparison between pathological metastasis or postoperative recurrence of the lymph node and other clinicopathological parameters might give misleading information. Therefore, we combined the metastatic stations and recurrent lymph node sites and classified them into five lymph node zones (Table S1). Lymph node metastasis in the cervical, upper mediastinal, middle mediastinal, lower mediastinal, and abdominal zones was detected in three (1.8%), six (3.6%), 10 (6.0%), 23 (13.7%), and 86 (51.2%) patients, respectively. Lymph node recurrence in the cervical, upper mediastinal, middle mediastinal, lower mediastinal, and abdominal zones was detected in one (0.6%), four (2.4%), one (0.6%), two (1.2%), and 12 (7.1%) patients, respectively. Details of metastatic stations and recurrent sites in the cervical, upper mediastinal, and middle mediastinal zones are summarized in Table 2. In the patient with recurrence at station No. 108, lymph node dissection of the middle mediastinal zone was not carried out during operation.

**Table 1:** Characteristics of patients with Siewert type II adenocarcinoma

Factors	Type II (n = 168) (%)
*Preoperative factors*	
Age (years, median) (range)	66 (24‐88)
Gender (Male/Female)	141 (83.9)/27 (16.1)
Barrett's carcinoma	31 (18.5)
Tumor length (mm, median) (range)	40 (10‐150)
Histology	
G1/G2	114 (67.9)
G3/4	54 (32.1)
*Surgical factors*	
Approach	
RTT	38 (22.6)
LTA	33 (19.6)
TH	97 (57.7)
Dissection of LN zone	
Cervical LN (yes)	13 (7.7)
Upper mediastinal LN (yes)	29 (17.3)
Middle mediastinal LN (yes)	74 (44.0)
Lower mediastinal LN (yes)	99 (58.9)
Abdominal LN (yes)	168 (100)
No. of dissected LN (mean ± SD)	35.6 ± 19.1
Operation time (min, median) (range)	306 (148‐724)
Bleeding (mL, median) (range)	359 (50‐1500)
Residual disease (R0/R1, 2)	156 (92.9) / 12 (7.1)
*Pathological factors*	
pT category	
pT1	62 (36.9)
pT2	42 (25.0)
pT3	57 (33.9)
pT4	6 (3.6)
pN category	
pN0	80 (47.6)
pN1	31 (18.5)
pN2	22 (13.1)
pN3	35 (20.8)
pM category	
pM0	161 (95.8)
pM1	6 (3.6)
pStage	
pI	71 (42.3)
pII	25 (14.9)
pIII	65 (38.7)
pIV	6 (3.6)
No. of LN metastasis (median) (range)	1.0 (0‐33)
Lymphatic invasion (positive)	94 (56.0)
Venous invasion (positive)	80 (47.6)
*Recurrence*	
LN (any)	15 (8.9)
Organ	30 (17.9)
Peritoneum	11 (6.5)

LN, lymph node; LTA, left‐sided thoracoabdominal incision; RTT, right‐sided transthoracic and abdominal incision; TH, transhiatal.

**Table 2:** Lymph node metastasis and recurrence in the cervical, upper mediastinal, and middle mediastinal zones in Siewert type II tumors

Lymph node zone	Metastasis		Recurrence	
	Station	Patients (n)	Station/site	Patients (n)
Cervical	Paraesophageal (No. 101)	2	Accessory nerve (No. 100ac)	1
	Supraclavicular (No.104)	3		
Upper mediastinal	Thoracic paraesophageal (105)	1	Pretracheal (106pre)	3
	Tracheobronchial (106tbL)	1	Recurrent nerve (106recL)	1
	Recurrent nerve (106rec)			
	Right side (106recR)	6		
	Left side (106recL)	2		
Middle mediastinal	Paraesophageal (108)	8	Paraesophageal (108)	1
	Subcarinal (107)	5		
	Main bronchus (109)			
	Right side (109R)	3		
	Left side (109L)	3		

### 3.2 Comparison of lymph node zone with metastasis or recurrence according to EIL in Siewert type II tumors

**Figure 1:**
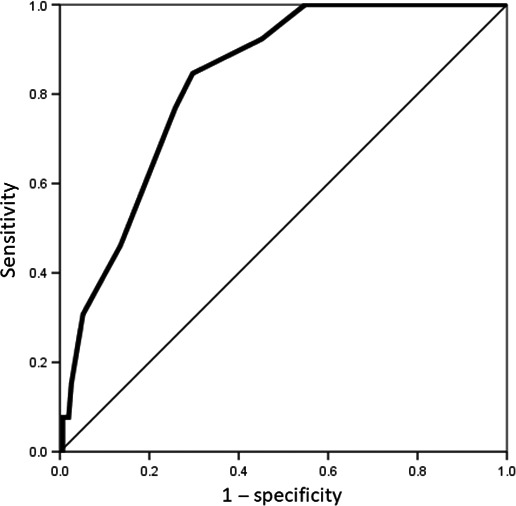
Receiver operating characteristic curves were generated for deciding the cut‐off value of esophageal invasion length (EIL) in Siewert type II tumors to predict the risk of mediastinal lymph node metastasis. Cut‐off value of EIL was determined as 25 mm with an area under the curve of 0.83 (sensitivity, 80.8%; specificity, 72.3%)

Distribution of overall metastasis or recurrence in the lymph node zones was assessed according to clinical EIL in patients with Siewert type II tumors (Table 3). Incidence of overall metastasis or recurrence was increased with the EIL of Siewert type II tumors, and this tendency was especially apparent in the mediastinal zones. Among patients with an EIL of <20 mm (n = 86), upper or middle mediastinal lymph node metastasis or recurrence occurred in one patient only (1%). In contrast, in patients with an EIL of more than 30 mm (n = 50), upper and middle mediastinal lymph node metastasis or recurrence occurred in 10 patients (20%). ROC curve for EIL in patients with Siewert type II tumor was generated to predict the rates of upper and middle mediastinal lymph node metastasis or recurrence, and the cut‐off EIL value was determined to be 25 mm with an area under the curve of 0.83 according to the ROC curve (sensitivity, 80.8%; specificity, 72.3%) (Figure 1). There were 118 patients whose EIL was less than or equal to 25 mm (≤25 mm EIL group) and 50 patients whose EIL was more than 25 mm (>25 mm EIL group).

**Table 3:** Lymph node zone with metastasis or recurrence according to EIL in Siewert type II tumor

Lymph node zone	EIL (mm)				
	0‐9 (n = 35) (%)	10‐19 (n = 51) (%)	20‐29 (n = 31) (%)	30‐39 (n = 23) (%)	40≤ (n = 27) (%)
Cervical					
Yes	0 (0.0)	0 (0.0)	0 (0.0)	2 (8.7)	2 (7.4)
No	35 (100)	51 (100)	31 (100)	21 (91.3)	25 (92.6)
Upper mediastinal					
Yes	0 (0.0)	1 (2.0)	1 (3.2)	2 (8.7)	4 (14.8)
No	35 (100)	50 (98.0)	30 (96.8)	21 (91.3)	23 (85.2)
Middle mediastinal					
Yes	0 (0.0)	1 (2.0)	1 (3.2)	3 (13.0)	6 (22.2)
No	35 (100)	50 (98.0)	30 (96.8)	20 (87.0)	21 (77.8)
Lower mediastinal					
Yes	2 (5.7)	5 (9.8)	4 (12.9)	9 (39.1)	5 (18.5)
No	33 (94.3)	46 (90.2)	27 (87.1)	14 (60.9)	22 (81.5)
Abdominal					
Yes	3 (8.6)	24 (47.1)	16 (51.6)	21 (91.3)	23 (85.2)
No	32 (91.4)	27 (52.9)	15 (48.4)	2 (8.7)	4 (14.8)

EIL, esophageal invasion length.

Patient characteristics were compared between the ≤25 mm EIL group and the >25 mm EIL group (Table 4). Tumor stages in the >25 mm EIL group were significantly more advanced than those in the ≤25 mm EIL group (*P* < .001). Lymphatic and venous invasions were significantly more frequent in the >25 mm EIL group (*P* < .001 and *P* = .002, respectively). Overall metastasis or recurrence was compared between the ≤25 mm EIL group and the >25 mm EIL group (Table 5). Rates of overall metastasis or recurrence in the cervical, upper, middle, lower mediastinal, and abdominal zones were significantly higher in the >25 mm EIL group than in the ≤25 mm EIL group (*P* = .007, *P* = .01, *P* < .001, *P* = .002, and *P* < .001, respectively).

**Table 4:** Characteristics of patients according to EIL in Siewert type II tumor

Factors	EIL (mm)			*P*
	Total (n = 168) (%)	≤25 (n = 118) (%)	>25 (n = 50) (%)	
*Preoperative factors*				
Age (years, median) (range)	66 (24‐88)	65 (24‐88)	66 (25‐82)	.82
Gender				
Male	141 (83.9)	100 (84.7)	41 (82.0)	.66
Female	27 (16.1)	18 (15.3)	9 (18.0)	
Barrett's carcinoma				
Yes	31 (18.5)	27 (22.9)	4 (8.0)	.023
No	137 (81.5)	91 (77.1)	46 (92.0)	
Tumor length (mm, median) (range)	40 (10‐150)	30 (10‐100)	60 (40‐150)	<.001
Histology				
G1/G2	114 (67.9)	85 (72.0)	29 (58.0)	.075
G3/G4	54 (32.1)	33 (28.0)	21 (42.0)	
cT category				
cT1	63 (37.5)	63 (53.4)	0 (0.0)	<.001
cT2/3	105 (62.5)	55 (46.6)	50 (100.0)	
cN category				
cN0	102 (60.7)	88 (74.6)	14 (28.0)	<.001
cN1/2/3	66 (39.3)	30 (25.4)	36 (72.0)	
cM category				
cM0	167 (99.4)	118 (100)	49 (98.0)	.30
cM1	1 (0.6)	0 (0.0)	1 (2)	
cStage				
cI/II	108 (64.3)	92 (78.0)	16 (32.0)	<.001
cIII/IV	60 (35.7)	26 (22.0)	34 (68.0)	
*Surgical factors*				
Approach				
RTT	38 (22.6)	11 (9.3)	27 (54.0)	<.001
LTA	33 (19.6)	20 (16.9)	13 (26.0)	
TH	97 (57.7)	87 (73.7)	10 (20.0)	
Dissection of LN zone				
Cervical LN				
Yes	13 (7.7)	2 (1.7)	11 (22.0)	<.001
No	155 (92.3)	116 (98.3)	39 (78.0)	
Upper mediastinal LN				
Yes	29 (17.3)	8 (6.8)	21 (42.0)	<.001
No	139 (82.7)	110 (93.2)	29 (58.0)	
Middle mediastinal LN				
Yes	74 (44.0)	39 (33.1)	35 (70.0)	<.001
No	94 (56.0)	79 (66.9)	15 (30.0)	
Lower mediastinal LN				
Yes	99 (58.9)	57 (48.3)	42 (84.0)	<.001
No	69 (41.1)	61 (51.7)	8 (16.0)	
No. of dissected LN (mean ± SD)	35.6 ± 19.1	31.6 ± 18.2	45.1 ± 18.2	<.001
Residual disease				
0	156 (92.9)	115 (97.5)	41 (82.0)	.001
1/2	12 (7.1)	3 (2.5)	9 (18.0)	
Morbidity				
Any				
Yes	53 (31.5)	35 (29.7)	18 (36.0)	.42
No	115 (68.5)	83 (70.3)	32 (64.0)	
Leakage				
Yes	15 (8.9)	11 (9.3)	4 (8.0)	.78
No	153 (91.1)	107 (90.7)	46 (92.0)	
Respiratory				
Yes	17 (10.1)	11 (9.3)	6 (12.0)	.60
No	151 (89.9)	107 (90.7)	44 (88.0)	
Wound infection				
Yes	8 (4.8)	5 (4.2)	3 (6.0)	.62
No	160 (95.2)	113 (95.8)	47 (94.0)	
Pancreatic fistula				
Yes	14 (8.3)	8 (6.8)	6 (12.0)	.26
No	154 (91.7)	110 (93.2)	44 (88.0)	
*Pathological factors*				
pT category				
pT1	62 (36.9)	60 (51.3)	2 (4.0)	<.001
pT2/3/4	105 (62.5)	57 (48.7)	48 (96.0)	
pN category				
pN0	80 (47.6)	73 (61.9)	7 (14.0)	<.001
pN1/2/3	88 (52.4)	45 (38.1)	43 (86.0)	
pM category				
pM0	161 (95.8)	116 (98.3)	45 (90.0)	.012
pM1	6 (3.6)	1 (0.8)	5 (10.0)	
pStage				
pI/II	96 (57.1)	83 (70.3)	13 (26.0)	<.001
pIII/IV	71 (42.3)	34 (28.9)	37 (74.0)	
No. of LN metastasis (median) (range)	1.0 (0‐33)	0 (0‐15)	5.5 (0‐33)	<.001
Lymphatic invasion				
Negative	73 (43.5)	64 (54.2)	9 (18.0)	<.001
Positive	94 (56.0)	53 (44.9)	41 (82.0)	
Venous invasion				
Negative	87 (51.8)	70 (59.3)	17 (34.0)	.002
Positive	80 (47.6)	47 (39.8)	33 (66.0)	
*Recurrence*				
LN (any)				
Yes	15 (8.9)	5 (4.2)	10 (20.0)	.001
No	152 (90.5)	112 (94.9)	40 (80.0)	
Organ				
Yes	30 (17.9)	17 (14.4)	13 (26.0)	.08
No	137 (81.5)	100 (84.7)	37 (74.0)	
Peritoneum				
Yes	11 (6.5)	7 (5.9)	4 (8.0)	.74
No	156 (92.9)	110 (93.2)	46 (92.0)	

EIL, esophageal invasion length; LN, lymph node; LTA, left‐sided thoracoabdominal incision; RTT, right‐sided transthoracic and abdominal incision; TH, transhiatal.

**Table 5:** Lymph node zone with metastasis and recurrence according to EIL in Siewert type II

Lymph node zone	Metastasis (%)			Recurrence (%)			Overall (Metastasis or recurrence) (%)		
	≤25 mm	>25 mm	*P*	≤25 mm	>25 mm	*P*	≤25 mm	>25 mm	*P*
Cervical									
Yes	0 (0.0)	3 (6.0)	.007	0 (0.0)	1 (2.0)	.30	0 (0.0)	4 (8.0)	.007
No	118 (100)	47 (94.0)		117 (99.2)	49 (98.0)		117 (100)	46 (92.0)	
Upper mediastinal									
Yes	0 (0.0)	6 (12.0)	<.001	2 (1.7)	2 (4.0)	.58	2 (1.7)	6 (12.0)	.01
No	118 (100)	44 (88.0)		115 (97.5)	48 (96.0)		115 (97.5)	44 (88.0)	
Middle mediastinal									
Yes	1 (0.8)	9 (18.0)	<.001	1 (0.8)	0 (0.0)	.65	2 (1.7)	9 (18.0)	<.001
No	117 (99.2)	41 (82.0)		116 (98.3)	50 (100)		115 (97.5)	41 (82.0)	
Lower mediastinal									
Yes	11 (9.3)	12 (24.0)	.01	0 (0.0)	2 (4.0)	.09	11 (9.3)	14 (28.0)	.002
No	107 (90.7)	38 (76.0)		117 (99.2)	48 (96.0)		106 (89.8)	36 (72.0)	
Abdominal									
Yes	43 (36.4)	43 (86.0)	<.001	6 (5.1)	6 (12.0)	.19	43 (36.4)	44 (88.0)	<.001
No	75 (63.6)	7 (14.0)		111 (94.1)	44 (88.0)		74 (62.7)	6 (12.0)	

EIL, esophageal invasion length.

### 3.3 Overall survival and risk assessment of mediastinal lymph node metastasis in Siewert type II tumors

**Figure 2:**
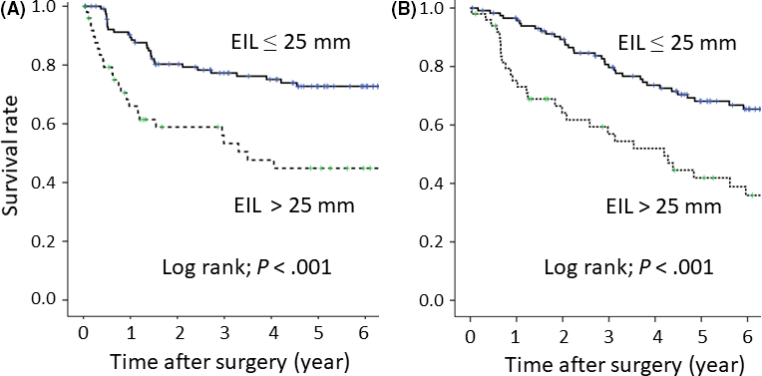
Kaplan ‐Meyer curves according to esophageal invasion length (EIL) in Siewert type II tumors. A, disease‐free survival of the >25 mm EIL group was significantly lower than that of the ≤25 mm EIL group (*P* < .001). B, Overall survival of the >25 mm EIL group was significantly lower than that of the ≤25 mm EIL group (*P* < .001)

**Figure 3:**
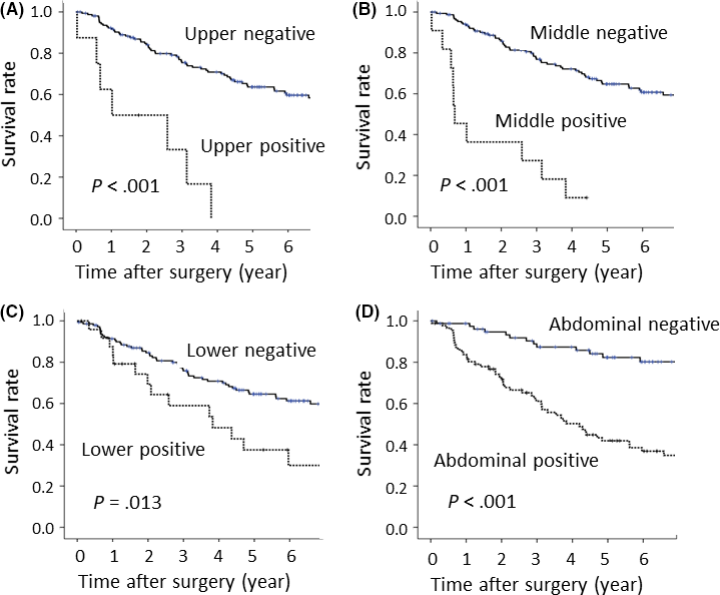
Overall survival of Siewert type II patients with overall metastasis or recurrence in each lymph node zone was significantly lower than that of Siewert type II patients without overall metastasis or recurrence. A, Upper mediastinal zone; *P* < .001. B, Middle mediastinal zone; *P* < .001. C, Lower mediastinal zone; *P* = .013. D, Abdominal zone; *P* < .001

Median duration of the follow‐up period for all the patients was 60 months. Estimated 5‐year disease‐free survival (DFS) rates for the ≤25 mm EIL group and the >25 mm EIL group were 67.1% and 41.3%, respectively (*P* < .001) (Figure 2A). Estimated 5‐year OS rates for the ≤25 mm EIL group and the >25 mm EIL group were 66.8% and 40.9%, respectively (*P* < .001) (Figure 2B). The 5‐year OS rates of the Siewert type II patients without overall metastasis or recurrence in the upper mediastinal, middle mediastinal, lower mediastinal, and abdominal zones were 64.7%, 62.8%, 64.5%, and 52.3%, respectively (Figure 3). In contrast, the rates of those with overall metastasis or recurrence in the upper mediastinal, middle mediastinal, lower mediastinal, and abdominal zones were 0%, 0%, 37.5%, and 41.9%, respectively, and these differences between patients with and those without overall metastasis or recurrence in each of the zones were significant for Siewert type II tumors (*P* < .001, *P* < .001, *P* = .013, and *P* < .001, respectively). Patients with Siewert type II tumors who had overall metastasis or recurrence in the cervical, upper, and middle mediastinal zones did not survive for more than 5 years after surgery. Efficacy indexes of the lower mediastinal lymph node and the abdominal lymph node were 5.6 and 21.7, respectively, for patients with Siewert type II tumors.

Univariate analyses showed that among preoperative parameters, EIL (≤25 mm vs >25 mm) (odds ratio, 9.09; 95% CI, 2.50‐33.3; *P* = .001), tumor length (odds ratio, 1.02; 95% CI, 1.00‐1.04; *P* = .018), and cN category (cN0 vs cN1/2/3) (odds ratio, 0.17; 95% CI, 0.04‐0.63; *P* = .008) were significantly correlated with overall metastasis or recurrence in the upper and middle mediastinal zones (Table S2). Multivariate analysis using a logistic regression method was used to assess the preoperative risk factors for overall metastasis or recurrence in the upper and middle mediastinal zones in Siewert type II tumor. Age, histology, EIL, tumor length, epicenter of the tumor, and cN were included in the model as preoperative parameters, but only an EIL of more than 25 mm was found to be an independent significant predictor of overall metastasis or recurrence in the upper and middle mediastinal zones in Siewert type II tumor (odds ratio, 8.85; 95% CI, 2.31‐33.3; *P* = .001).

## 4 DISCUSSION

The present study indicated that overall metastasis or recurrence in the upper and middle mediastinal zones is associated with EIL in patients with Siewert type II tumors, and an EIL of more than 25 mm might be a preoperative predictor of overall metastasis or recurrence in the upper and middle mediastinal zones. Few studies have investigated the preoperative risk factors for mediastinal lymph node metastasis or recurrence in patients with Siewert type II tumors. Indeed, the correct identification of patients with a high risk of upper and middle mediastinal lymph node metastasis would help in decisions regarding an appropriate surgical approach (transthoracic or transhiatal) and en‐bloc mediastinal lymph node dissection. In the present study, we showed that the EIL was significantly associated with upper and middle mediastinal lymph node metastasis or recurrence. As is well known, the risk of lymph node metastasis increases considerably when the tumor has invaded to the submucosal layer of the esophageal wall because of the enormously rich lymphatic flow beneath the esophageal muscularis mucosal layer.^19^, ^20^ This means that a thorough reginal lymph node dissection is required even in patients with Siewert type II tumors with submucosal invasion (T1b). However, because of a limitation of the accurate preoperative diagnosis of lymph node metastasis and the frequent discrepancy between clinical diagnoses and pathological findings, the surgical procedures for Siewert type II tumors unfortunately still depend on individual surgeons or institutes. Moreover, with the dawn of the era of neoadjuvant chemotherapy or chemoradiotherapy for EGJ adenocarcinoma, a novel preoperative indicator for mediastinal lymph node metastasis is needed. The EIL can be determined during routine diagnostic examinations, such as endoscopy or esophagogastrography, and might help to determine whether a transthoracic approach should be used and whether upper and middle mediastinal lymph node dissection should be carried out.

In the present study, we determined the cut‐off value of EIL as 25 mm, and an EIL of more than 25 mm was a significant predictor of upper and middle mediastinal lymph node metastasis. The latest TNM Classification, the 8th edition of the International Union Against Cancer TNM Classification of Malignant Tumors (UICC 2017),^5^ has changed the concept of EGJ tumors. In the new classification rules, a tumor with an epicenter within 2 cm of the EGJ and also extending into the esophagus is classified and staged using an esophageal scheme, and cancers involving the EGJ with an epicenter of within the proximal 2 cm of the cardia (Siewert types I/II) are to be staged as esophageal cancer. These rules suggest that the therapeutic strategy, including surgical procedures, might differ depending on whether the proximal edge of Siewert type II tumor extends beyond a point 2 cm proximal from the EGJ. In a nation‐wide survey, Yamashita et al^3^ investigated mediastinal lymph node metastasis of EGJ cancer having its epicenter within 2 cm proximal or 2 cm distal to the EGJ and its diameter of less than 4 cm. They showed that, for the patients with esophagus‐predominant EGJ adenocarcinoma, the incidence of lymph node metastasis was 0%, 0.4%, and 1.7% in the cervical, upper mediastinal, and middle mediastinal zones, respectively. They also showed that the incidence of lymph node recurrence was approximately 1% in the cervical, upper mediastinal, and middle mediastinal zones. These very low incidences of lymph node metastasis or recurrence in the cervical, upper mediastinal, and middle mediastinal zones can be explained from the tumor location within 2 cm proximal to the EGJ: this means that an EIL of the tumor is <2 cm. Kurokawa et al^26^ investigated the correlation between the EIL and mediastinal lymph node metastasis. Although their study included 14% of patients who received neoadjuvant chemotherapy and excluded patients diagnosed as pT1, they showed that an EIL of more than 3 cm was a predictor of upper and middle mediastinal lymph node metastasis. In our study, none of the patients received neoadjuvant therapies and cT1 patients were included; as a result, a point 25 mm from the EGJ was considered the border for an increased risk of upper and middle mediastinal lymph node metastasis or recurrence. Our cut‐off value was almost identical to the border of the new TNM classification. Based on these findings, the EIL might be useful for deciding on an appropriate surgical procedure: an upper and middle mediastinal lymph node dissection carried out by RTT should be recommended when the EIL of Siewert type II tumor is more than 25 mm. In contrast, upper and middle mediastinal lymph node metastasis or recurrence was rare in the ≤25 mm EIL group. However, lower mediastinal lymph node metastasis or recurrence was found in about 10% of patients in the ≤25 mm EIL group. Therefore, TH with lower mediastinal lymph node dissection, and not RTT, is recommended for the ≤25 mm EIL group.

The overall survival rate in the >25 mm EIL group was dismal. Recurrence is a considerable concern regarding the survival of patients with Siewert type II tumors.^27^, ^28^ Lymph node recurrence occurred in 15 (8.9%) patients with Siewert type II tumor. Among them, there were six (3.6%) patients whose lymph node recurrences occurred in the cervical and mediastinal lymph node zones, and almost all lymph node recurrences occurred at stations where we do not carry out dissection even in RTT with cervical lymphadenectomy. Although this might indicate that the recurrent lymph node showed different biological behavior from the pathological lymph node metastasis after surgery, we used parameters such as overall metastasis or recurrence in the lymph node zone, because of the surgical bias, including the extent of lymph node dissection. In fact, none of the patients with metastasis or recurrence in the upper and middle mediastinal zones survived for more than 5 years after surgery among the patients with Siewert type II tumors. These findings strongly indicated that a multimodal therapeutic strategy should be considered to improve the survival of patients with an EIL of more than 25 mm. In contrast, the ≤25 mm EIL group showed a promising survival outcome. This was, in part, associated with the low incidence of mediastinal lymph node metastasis or recurrence. Consequently, surgery alone might be recommended as a therapeutic strategy for the ≤25 mm EIL group if no evidence of clinical lymph node metastasis is present prior to treatment.

Besides the biases arising from the different surgical procedures and the lack of treatment allocation, the present study had several other limitations. We determined the cut‐off value and carried out further analyses. However, the study was carried out retrospectively; therefore, the robustness of the cut‐off value should be investigated in future analyses. Moreover, the sample size was unequally distributed into individual groups. Usefulness of EIL in patients with a large hiatal hernia also remains unknown. Therefore, to address these concerns, a large number of patients with an EIL of more than 25 mm is required to investigate the exact role of EIL in Siewert type II tumors.

EIL in patients with Siewert type II tumors could be a clinical surrogate for upper and middle mediastinal lymph node metastasis or recurrence. To the best of our knowledge, this is the first study to show a difference in OS according to EIL among patients with Siewert type II tumors. Although the efficacy of the upper and middle mediastinal lymph node dissection remains to be further investigated, EIL might contribute to establishing an appropriate therapeutic strategy in patients with Siewert type II tumors.

## DISCLOSURE

Ethical Statement: The protocol for this retrospective study has been approved by the institutional review board of the National Cancer Center Hospital (Approved No. 2017‐061). This work followed the guidelines set forth in the Helsinki Declaration of 1975, as revised in 2000, concerning Human and Animal Rights. This article does not contain human or animal subjects performed by any authors.

Conflicts of Interest: Authors declare no conflicts of interest for this article.
