# Supplementary material for: Clinical significance of esophageal invasion length for the prediction of mediastinal lymph node metastasis in Siewert type II adenocarcinoma: A retrospective single‐institution study
Source: Ann Gastroenterol Surg. 2018 Apr 10;2(3):187–96. doi: 10.1002/ags3.12069 (PMC5980392; doi:10.1002/ags3.12069)
Supplement: Supplementary file 1 [file AGS3-2-187-s001.docx]

**Supplemental Table 1** Lymph node zone with metastasis and recurrence in Siewert type II tumor

| Lymph node zone |  | Metastasis (%) |  | Recurrence (%) |  | Overall (metastasis or recurrence) (%) |
| --- | --- | --- | --- | --- | --- | --- |
| Cervical |  |  |  |  |  |  |
| Yes |  | 3 (1.8) |  | 1 (0.6) |  | 4 (2.4) |
| No |  | 165 (98.2) |  | 166 (98.8) |  | 163 (97.0) |
| Upper mediastinal |  |  |  |  |  |  |
| Yes |  | 6 (3.6) |  | 4 (2.4) |  | 8 (4.8) |
| No |  | 162 (96.4) |  | 163(97.0) |  | 159 (94.6) |
| Middle mediastinal |  |  |  |  |  |  |
| Yes |  | 10 (6.0) |  | 1 (0.6) |  | 11 (6.5) |
| No |  | 158 (94.0) |  | 166 (98.8) |  | 156 (92.9) |
| Lower mediastinal |  |  |  |  |  |  |
| Yes |  | 23 (13.7) |  | 2 (1.2) |  | 25 (14.9) |
| No |  | 145 (86.3) |  | 165 (98.2) |  | 142 (84.5) |
| Abdominal |  |  |  |  |  |  |
| Yes |  | 86 (51.2) |  | 12 (7.1) |  | 87 (51.8) |
| No |  | 82 (48.8) |  | 155 (92.3) |  | 80 (47.6) |
