# Supplementary material for: Clinical significance of esophageal invasion length for the prediction of mediastinal lymph node metastasis in Siewert type II adenocarcinoma: A retrospective single‐institution study
Source: Ann Gastroenterol Surg. 2018 Apr 10;2(3):187–96. doi: 10.1002/ags3.12069 (PMC5980392; doi:10.1002/ags3.12069)
Supplement: Supplementary file 2 [file AGS3-2-187-s002.docx]

**Supplemental Table 2** Univariate and multivariate analysis for prediction of overall metastasis or recurrence in the upper and middle mediastinal zone

|  |  | Univariate analysis | | | | |  | Multivariate analysis | | | | |
| --- | --- | --- | --- | --- | --- | --- | --- | --- | --- | --- | --- | --- |
| Factors |  | Odds |  | 95% CI |  | *P* |  | Odds |  | 95% CI |  | *P* |
| Age (>65 vs. ≤65) |  | 2.50 |  | 0.73‒8.45 |  | 0.14 |  |  |  |  |  |  |
| Gender (Male vs. Female) |  | 2.44 |  | 0.30‒19.6 |  | 0.40 |  |  |  |  |  |  |
| Barrett carcinoma |  | 1.28 |  | 0.27‒6.07 |  | 0.76 |  |  |  |  |  |  |
| Tumor length |  | 1.02 |  | 1.00‒1.04 |  | 0.018 |  |  |  |  |  |  |
| Histology (G1/2 vs. G3/4) |  | 0.38 |  | 0.12‒1.18 |  | 0.09 |  |  |  |  |  |  |
| EIL (>25 vs. ≤25 mm) |  | 9.09 |  | 2.50‒33.3 |  | 0.001 |  | 8.85 |  | 2.31‒33.3 |  | 0.001 |
| Epicenter (G vs. E) |  | 0.29 |  | 0.06‒1.37 |  | 0.12 |  |  |  |  |  |  |
| cT category (T1 vs T2/3) |  | 0.00 |  | 0.00‒ |  | 0.997 |  |  |  |  |  |  |
| cN category (N0 vs 1/2/3) |  | 0.17 |  | 0.04‒0.63 |  | 0.008 |  |  |  |  |  |  |

EIL, esophageal invasion length; G, gastric; E, esophagus.
